# Antibiotic prescription patterns in the empiric therapy of severe sepsis: combination of antimicrobials with different mechanisms of action reduces mortality

**DOI:** 10.1186/cc11869

**Published:** 2012-11-18

**Authors:** Ana Díaz-Martín, María Luisa Martínez-González, Ricard Ferrer, Carlos Ortiz-Leyba, Enrique Piacentini, Maria Jesus Lopez-Pueyo, Ignacio Martín-Loeches, Mitchell M Levy, Antoni Artigas, José Garnacho-Montero, for the Edusepsis Study Group

**Affiliations:** 1 Intensive Care Unit, Critical Care and Emergency Department, Virgen del Rocío University Hospital, Avda. Manuel Siurot s/n, Seville 41013, Spain; 2 Instituto de Biomedicina de Sevilla (IBIS), Virgen del Rocío University Hospital/CSIC/Seville University, 41013 Seville, Spain; 3 Spanish Network for Research in Infectious Disease (REIPI), Virgen del Rocío University Hospital, Avda. Manuel Siurot, s/n. 41013, Seville, Spain; 4 Critical Care Center, Sabadell Hospital, Autonomous University of Barcelona, Corporació Sanitaria Universitària Parc Taulí, 08208 Sabadell, Spain; 5 Intensive Care Unit, Mútua Terrassa University Hospital, University of Barcelona, Plaça Dr, Robert 5, Terrassa, 08221, Barcelona, Spain; 6 CIBER-Enfermedades Respiratorias; 7 Intensive Care Unit, General Yagüe Hospital, Avda del Cid Campeador, 96 09005 Burgos, Spain; 8 Medical Intensive Care Unit, Rhode Island Hospital/Brown University, 593 Eddy St,, Providence, RI 02903, USA

## Abstract

**Introduction:**

Although early institution of adequate antimicrobial therapy is lifesaving in sepsis patients, optimal antimicrobial strategy has not been established. Moreover, the benefit of combination therapy over monotherapy remains to be determined. Our aims are to describe patterns of empiric antimicrobial therapy in severe sepsis, assessing the impact of combination therapy, including antimicrobials with different mechanisms of action, on mortality.

**Methods:**

This is a Spanish national multicenter study, analyzing all patients admitted to ICUs who received antibiotics within the first 6 hours of diagnosis of severe sepsis or septic shock. Antibiotic-prescription patterns in community-acquired infections and nosocomial infections were analyzed separately and compared. We compared the impact on mortality of empiric antibiotic treatment, including antibiotics with different mechanisms of action, termed different-class combination therapy (DCCT), with that of monotherapy and any other combination therapy possibilities (non-DCCT).

**Results:**

We included 1,372 patients, 1,022 (74.5%) of whom had community-acquired sepsis and 350 (25.5%) of whom had nosocomial sepsis. The most frequently prescribed antibiotic agents were β-lactams (902, 65.7%) and carbapenems (345, 25.1%). DCCT was administered to 388 patients (28.3%), whereas non-DCCT was administered to 984 (71.7%). The mortality rate was significantly lower in patients administered DCCTs than in those who were administered non-DCCTs (34% versus 40%; *P *= 0.042). The variables independently associated with mortality were age, male sex, APACHE II score, and community origin of the infection. DCCT was a protective factor against in-hospital mortality (odds ratio (OR), 0.699; 95% confidence interval (CI), 0.522 to 0.936; *P *= 0.016), as was urologic focus of infection (OR, 0.241; 95% CI, 0.102 to 0.569; *P *= 0.001).

**Conclusions:**

β-Lactams, including carbapenems, are the most frequently prescribed antibiotics in empiric therapy in patients with severe sepsis and septic shock. Administering a combination of antimicrobials with different mechanisms of action is associated with decreased mortality.

## Introduction

Sepsis is a prevalent disorder and one of the main causes of death among hospitalized patients. Treating sepsis is associated with high costs; however, despite advances in medical practice, the mortality rate of sepsis has not declined in recent decades [1]. In Spain, the incidence of severe sepsis is 104 cases per 100,000 adult residents per year, and related in-hospital mortality is 20.7%; the incidence of septic shock is 31 cases per 100,000 adult residents per year, and related in-hospital mortality is 45.7% [2]. Sepsis present at intensive care unit (ICU) admission and ICU-acquired sepsis clearly differ in the types of patients affected, the sources of infection, the microorganisms responsible, and the prognosis [3].

Diverse studies have confirmed that the prompt institution of antimicrobial therapy active against the causative pathogen is lifesaving in patients with severe sepsis [4,5]. The Surviving Sepsis Campaign strongly recommends initiating antibiotic therapy within the first hour of recognition of severe sepsis, after suitable samples have been obtained for cultures [6].

Nevertheless, although antibiotic therapy is the cornerstone in the treatment of sepsis, the optimal antimicrobial strategy has not been defined. Few data are available about antibiotic prescription patterns used most in severe sepsis.

Furthermore, the advantages and disadvantages of combination therapy compared with monotherapy are controversial, and studies comparing the two approaches have mainly been limited to bacteremia, pneumonia, or serious *Pseudomonas aeruginosa *infections [7-9]. Importantly, a recent retrospective study concluded that certain combinations of antimicrobials, including antimicrobials with different targets, improve survival in patients with septic shock [10].

We present a secondary analysis of the Edusepsis study, which enrolled all patients with severe sepsis and septic shock admitted to the participating ICUs during 2 months in 2005 and 4 months in 2006. Our aims are (a) to describe the patterns of empiric antimicrobial therapy, analyzing the differences between community-acquired and nosocomial infections; and (b) to compare the impact on mortality of combination therapy, including at least two antimicrobials with different mechanisms of action, with that of monotherapy and other combinations of antimicrobials.

## Materials and methods

### Design of the study

We conducted a secondary analysis of the Edusepsis study, a Spanish national multicenter before-and-after study involving 77 ICUs [11]. In this study, carried out between November 2005 and 2007, data were collected before and after a 2-month educational intervention based on the Surviving Sepsis Campaign guidelines; this approach to improving treatment of severe sepsis is cost-effective [12]. Each participating centers' research and ethical-review boards approved the study, and patients remained anonymous. The need for informed consent was waived in view of the observational and anonymous nature of the study.

The study included all patients in these ICUs with severe sepsis or septic shock. The study design is described in detail elsewhere [11]. In brief, severe sepsis was defined as sepsis associated with organ dysfunction unexplained by other causes. Septic shock was defined as sepsis associated with systolic blood pressure <90 mm Hg, mean arterial pressure <65 mm Hg, or a reduction in systolic blood pressure >40 mm Hg from baseline despite adequate volume resuscitation. Patients in whom the onset of severe sepsis could not be determined were excluded from the analysis. The approach to data collection and the quality-control measures to assure data reliability also are described elsewhere [11,12].

### Variables

The following variables were recorded: demographic characteristics (age and gender), types of patients (medical, trauma, emergency surgery, elective surgery), sources of infection, location at sepsis acquisition (community-acquired or nosocomial infection), and baseline lactate level and organ dysfunction at sepsis diagnosis. Severity of illness was evaluated by the Acute Physiology and Chronic Health Evaluation (APACHE) II score, considering the worst reading in the first 24 hours in the ICU [13]. All patients were followed up until death or hospital discharge. The primary outcome variable was in-hospital mortality.

### Antimicrobial therapy

The antimicrobial therapy prescribed at the diagnosis of severe sepsis and the time from severe sepsis presentation to antibiotic administration were recorded. To facilitate subsequent analysis, antimicrobial agents were grouped into eight antibiotic families: β-lactams (except carbapenems), carbapenems, quinolones, macrolides, aminoglycosides, anti-gram-positive antibiotics (vancomycin, teicoplanin, and linezolid), antifungal agents, and other antimicrobial agents (including antiviral and tuberculostatic agents). Data for community-acquired and nosocomial infections also were analyzed separately. We also compared the clinical characteristics of patients that received different-class combination therapy (DCCT) with those of patients that received any other antimicrobial therapy (non-DCCT).

DCCT was defined as the concomitant use of two or more antibiotics of different mechanistic classes, as recently defined by Kumar *et al. *[10], specifically β-lactams or carbapenems with aminoglycosides, fluoroquinolones, or macrolides/clindamycin. Monotherapy or any other combination therapy was considered non-DCCT for this analysis.

To assess the impact of DCCT on mortality, we analyzed only patients who received the first dose of antimicrobial within the first 6 hours after severe sepsis presentation.

### Statistical analysis

Discrete variables were expressed as frequencies (percentage), and continuous variables, as means and standard deviations (SDs), unless stated otherwise; all statistical tests were two-sided. Differences in categoric variables were calculated by using χ^2 ^tests or Fisher Exact test, and differences in continuous variables were calculated by using the Mann-Whitney *U *or Kruskal-Wallis test, as appropriate.

Backward logistic regression was used to assess the factors independently associated with in-hospital mortality. To avoid spurious associations, variables entered in the regression models were those with a relation in univariate analysis (*P *≤ 0.05) or a plausible relation with the dependent variable. SPSS for Windows 20.0 (SPSS, Chicago, IL, USA) was used for all statistical analyses.

## Results and discussion

### Descriptive analysis

The Edusepsis study included 2,796 patients with severe sepsis or septic shock; we analyzed the 1,372 patients that received antibiotic therapy in the first 6 hours from the diagnosis of sepsis, of whom 1,022 (74.5%) had community-acquired sepsis and 350 (25.5%) had nosocomial sepsis. Table 1 shows the study group's main demographics, APACHE II scores, levels of lactate, and diagnoses on admission.

**Table 1:** Demographic and clinical characteristics of the patients

	Global *n *= 1,372	Community-acquired *n *= 1,022 (74.5%)	Nosocomial *n *= 350 (25.5%)	*P*
General data				

Sex (male)	837 (61%)	623 (61%)	214 (61%)	0.999
Age (years)	62.24 ± 16.22	62.00 ± 16.65	62.93 ± 14.87	0.354
APACHE II	21.44 ± 7.54	21.09 ± 7.49	22.45 ± 7.61	0.004
Lactate (m***M***)	35.56 ± 26.94	36.76 ± 27.68	32.06 ± 24.37	0.020

Diagnosis on admission				

Medical	893 (65.4%)	734 (72%)	159 (45.8%)	
Trauma	25 (1.8%)	8 (0.8%)	17 (4.9%)	<0.001
Emergency surgery	382 (28%)	256 (25.1%)	126 (36.3%)	
Elective surgery	66 (4.86%)	21 (2.1%)	45 (13%)	

Type of infection				

Pneumonia	502 (36.6%)	362 (35.4%)	140 (40%)	
Abdominal	390 (28.4%)	270 (26.4%)	120 (34.3%)	
Urologic	182 (13.3%)	163 (15.9%)	19 (5.4%)	
Meningitis	50 (3.6%)	47 (4.6%)	3 (0.9%)	<0.001
SSTI	54 (3.9%)	46 (4.5%)	8 (2.3%)	
Catheter	24 (1.7%)	6 (0.6%)	18 (5.1%)	
Others	138 (3.1%)	108 (10.6%)	30 (8.6%)	
More than one focus	32 (2.3%)	20 (2.0%)	12 (3.4%)	

Organ failure				

Hemodynamic	1,129 (82.3%)	845 (82.7%)	284 (81.1%)	0.517
Respiratory	880 (64.1%)	641 (62.7%)	239 (68.3%)	0.062
Renal	,1006 (73.3%)	754 (73.8%)	252 (72.0%)	0.529
Hepatic	238 (17.3%)	176 (17.2%)	62 (17.7%)	0.870
Hematologic	344 (25.1%)	266 (26.0%)	78 (22.3%)	0.175
Coagulation	502 (36.6%)	394 (38.6%)	108 (30.9%)	0.010

Mortality	526 (38.3%)	356 (34.8%)	170 (48.6%)	<0.001

The most frequent sources of sepsis were pneumonia (*n *= 502; 36.6%), followed by abdominal infection (*n *= 390; 28.48%), urinary tract infection (*n *= 182; 13.3%), central nervous system infection (*n *= 50; 3.6%), skin or soft-tissue infection (*n *= 54; 3.9%), and catheter-related infection (*n *= 24; 1.7%).

### Antimicrobial treatments prescribed

The most frequently prescribed antibiotic agents were β-lactams (*n *= 902; 65.7%), carbapenems (*n *= 345; 25.1%), and quinolones (*n *= 282; 20.6%). Table 2 presents the data for the entire group of patients who received empiric antibiotic therapy within 6 hours of admission, and for the groups of patients with community-acquired (*n *= 1,022; 74.5%) and nosocomial infections (*n *= 350; 25.5%).

**Table 2:** Antibiotic distribution in the entire cohort and in patients with community-acquired and nosocomial sepsis

Antibiotics	Global *n *= 1,372	Community-acquired *n *= 1,022 (74.5%)	Nosocomial *n *= 350 (25.5%)	*P*
β-lactams	902 (65.7%)	708 (69.3%)	194 (55.4%)	<0.001
Carbapenems	345 (25.1%)	218 (21.3%)	127 (36.3%)	<0.001
Quinolones	282 (20.6%)	241 (23.6%)	41 (11.7%)	<0.001
Aminoglycosides	183 (13.3%)	114 (11.2%)	69 (19.7%)	<0.001
Macrolides	60 (4.4%)	54 (5.3%)	6 (1.7%)	0.004
Anti-gram-positive	161 (11.7%)	96 (9.4%)	65 (18.6%)	<0.001
Antifungals	38 (2.8%)	20 (2.0%)	18 (5.1%)	0.004
Others	151 (11.0%)	111 (10.9%)	40 (11.4%)	0.767

The distribution of the antibiotics prescribed for community-acquired infections was similar to that for the overall group, with predominance of β-lactams (*n *= 708; 69.3%), quinolones (*n *= 241; 23.6%), and carbapenems (*n *= 218; 21.3%), , whereas in the group with nosocomial infections, although β-lactams were also the most-used treatment (*n *= 194; 55.4%), carbapenems were second (*n *= 127; 36.3%), followed by aminoglycosides (*n *= 69; 19.7%) and anti-gram-positive agents (*n *= 65; 18.6%). Macrolides and quinolones were more frequently used in community-acquired sepsis than in nosocomial sepsis (see Table 2).

### DCCT and non-DCCT groups

DCCTs were administered to 388 patients (28.3%), and non-DCCTs, to 984 (71.7%). Table 3 shows the demographic characteristics, diagnosis at admission, incidence of associated organ failure, and sources of infection of patients in the DCCT and non-DCCT groups. Sex distribution, age, APACHE II score, and lactate levels were very similar in the two groups.

**Table 3:** Comparisons of patients treated with DCCT or non-DCCT

	DCCT group *n *= 388 (28.3%)	Non-DCCT group *n *= 984 (71.7%)	*P*
General data			

Sex (male)	247 (63.7%)	590 (60%)	0.219
Age (years)	60.88 ± 16.79	62.78 ± 15.96	0.057
APACHE II	21.35 ± 7.43	21.47 ± 7.58	0.790
Lactate (m***M***)	36.37 ± 26.99	35.22 ± 26.93	0.578

Diagnosis on admission			

Medical	310 (79.9%)	583 (59.6%)	<0.001
Trauma	3 (0.8%)	22 (2.2%)	0.074
Emergency surgery	59 (15.2%)	323 (33%)	<0.001
Elective surgery	16 (4.1%)	50 (5.1%)	0.487

Type of infection			

Abdominal	56 (14.4%)	334 (33.9%)	<0.001
Urologic	44 (11.3%)	138 (14%)	0.216
Meningitis	5 (1.3%)	45 (4.6%)	0.002
Skin and/or soft-tissue	6 (1.5%)	48 (4.9%)	0.003
Catheter	4 (1%)	20 (2%)	0.256
Others	31 (8%)	107 (10.9%)	0.134
More than one focus	13 (3.4%)	19 (1.9%)	0.162

Organ failure			

Number of organ failures	2.98 ± 1.26	2.98 ± 1.25	0.955
Hemodynamic	319 (82.2%)	810 (82.3%)	0.999
Respiratory	289 (74.5%)	591 (60.1%)	<0.001
Renal	264 (68%)	742 (75.4%)	0.007
Hepatic	61 (15.7%)	177 (18%)	0.343
Hematologic	94 (24.2%)	250 (25.4%)	0.679
Coagulation	131 (33.8%)	371 (37.7%)	0.191
Mortality	132 (34%)	394 (40%)	0.042

Significant differences between the two groups were found in diagnosis at admission and source of infection. In the DCCT group, the percentage of patients with medical diagnoses was higher (79.9% versus 59.6%; *P *< 0.001) and the percentage with emergency surgical diagnoses was lower (15.2% versus 33%; *P *< 0.001). The most common source of sepsis was pneumonia in the DCCT group (59% versus 27.7%; *P *< 0.001) and abdominal infection in the non-DCCT group (14.4% versus 33.9%; *P *< 0.001).

Although the median number of organ failures was the same in both groups, significant differences were noted in the organ-failure distribution: respiratory failure was more common in the DCCT group (74.5% versus 60.1%; *P *< 0.001) and renal failure was more common in the non-DCCT group (68% versus 75.4%; *P *= 0.007).

In the DCCT group, the most frequently used agents were β-lactams (*n *= 320; 82.5%), followed by quinolones (*n *= 186; 47.9%), aminoglycosides (*n *= 158; 40.7%), and carbapenems (*n *= 76; 19.6%) (Table 4). These agents were used in the following combinations: (a) a β-lactam plus an aminoglycoside or a quinolone or a macrolide (*n *= 312; 80.4%); the most common combination in this group was a β-lactam plus a quinolone (*n *= 163; 52.2%); (b) a carbapenem plus an aminoglycoside or a quinolone or a macrolide (*n *= 68; 17.5%); the most common combination in this group was a carbapenem plus an aminoglycoside (*n *= 46; 67.6%); and (c) a β-lactam plus a carbapenem (*n *= 8; 2.1%), usually associated with an aminoglycoside (*n *= 6; 75.0%) (data not shown in table). It is noteworthy that DCCT consisted only of a β-lactam or carbapenem plus a macrolide and/or an aminoglycoside and/or a quinolone in 311 (80%) patients; thus, other antimicrobials (antifungals, anti-gram-positive agents, and so on) were also administered in DCCT in only 75 (20%) (data not shown).

**Table 4:** Antibiotic prescription in patients treated with DCCT or non-DCCT

Antibiotics	Non-DCCT group *n *= 984 (71.7%)	DCCT group *n *= 388 (28.3%)	*P*
β-Lactams	582 (59.1%)	320 (82.5%)	<0.001
Carbapenems	269 (27.3%)	76 (19.6%)	0.003

Quinolones	96 (9.8%)	186 (47.9%)	<0.001
Aminoglycosides	25 (2.5%)	158 (40.7%)	<0.001
Macrolides	7 (0.7%)	53 (13.7%)	<0.001
Anti-gram-positive	120 (12.2%)	41 (10.6%)	0.456
Antifungals	21 (2.1%)	17 (4.4%)	0.028
Others	121 (12.3%)	30 (7.7%)	0.016

### Predictors of mortality

In the univariate analysis, factors significantly associated with mortality were gender, age, APACHE II score, lactate levels, source of infection, and DCCT (Table 5). Mortality was significantly lower in the DCCT group (34.0% versus 40%; *P *= 0.042). In the multivariate analysis (Table 6), including the variables that were significantly associated with mortality in the univariate analysis, higher age (OR, 1.023; 95% CI, 1.014 to 1.032; *P *< 0.001), male sex (OR, 1.350; 95% CI, 1.041 to 1.750; *P *= 0.024), higher APACHE II score (OR, 1.099; 95% CI, 1.099 to 1.141; *P *< 0.001), and community-acquired infection (OR, 1.487; 95% CI, 1.119 to 1.974; *P *= 0.006) was associated with higher mortality, whereas urologic focus of infection (OR, 0.241; 95% CI, 0.102 to 0.569; *P *= 0.001) and DCCT were associated with lower mortality (OR, 0.699; 95% CI, 0.522 to 0.936; *P *= 0.016).

**Table 5:** Univariate analysis of factors associated with in-hospital mortality

	Global *n *= 1,372	Survivors *n *= 846 (61.7%)	Nonsurvivors *n *= 526 (38.3%)	*P*
General data				

Sex (male)	837 (61.0%)	489 (57.8%)	348 (66.2%)	0.002
Age (years)	62.2 ± 16.2	59.80 ± 16.81	66.16 ± 14.39	<0.001
APACHE II	21.4 ± 7.5	19.20 ± 6.86	25.09 ± 7.17	<0.001
Lactate (m***M***)	35.6 ± 26.9	31.09 ± 22.54	43.09 ± 31.69	<0.001

Type of infection				

Pneumonia	502 (36.6%)	289 (34.2%)	213 (40.5%)	
Abdominal	390 (28.4%)	228 (27%)	162 (30.8%)	
Urologic	181 (13.3%)	142 (16.8%)	40 (7.6%)	
Meningitis	50 (3.6%)	40 (4.7%)	10 (1.9%)	<0.001
Skin and soft-tissue	54 (3.9%)	37 (4.4%)	17 (3.2%)	
Catheter	24 (1.7%)	15 (1.8%)	9 (1.7%)	
Others	138 (10.1%)	78 (9.2%)	60 (11.4%)	
More than one focus	32 (2.3%)	17 (2%)	15 (2.9%)	

Community acquired	1,022 (74.5%)	666 (78.7%)	356 (67.7%)	<0.001

DCCT	388 (28.3%)	256 (30.3%)	132 (25.1%)	0.042

**Table 6:** Multivariate analysis of risk factors for mortality

Factors	OR	CI (95%)	*P*
Age (years)	1.023	(1.014-1.032)	<0.001
Sex (male)	1.350	(1.041-1.750)	0.024
APACHE II	1.099	(1.099-1.141)	<0.001
Community-acquired	1.487	(1.119-1.974)	0.006
DCCT	0.699	(0.522-0.936)	0.016

Focus of infection			

Pneumonia	0.784	(0.358-1.718)	0.543
Abdominal	0.595	(0.269-1.317)	0.200
Urologic	0.241	(0.102-0.569)	0.001
Meningitis	0.357	(0.122-1.046)	0.060
Skin and soft-tissue	0.424	(0.157-1.141)	0.089
Catheter	0.441	(0.135-1.445)	0.177
Others	0.772	(0.330-1.806)	0.551
More than one focus	1		

For the DCCT-combination treatments associated with reductions in mortality, the results of the analysis, excluding patients who died within the first 6 hours, were similar to the results including these patients; hence, no evidence of immortal bias was found in our results.

## Discussion

This secondary analysis of the Edusepsis study reveals interesting data about the patterns of antibiotic prescription in patients with severe sepsis and septic shock and about the characteristics of patients receiving combination therapy, including antimicrobials, with different mechanisms of action (DCCTs) versus those receiving either monotherapy or any other combinations of antimicrobials (non-DCCTs). Our study confirms the increased survival in patients administered DCCTs (β-lactams plus aminoglycosides, quinolones, or macrolides/clindamycin) within the first 6 hours of severe sepsis presentation. We excluded patients that received antimicrobial therapy after 6 hours of severe sepsis diagnosis from this analysis, because strong evidence indicates that early administration increases survival in patients with severe sepsis or septic shock [4,5,10].

Appropriate empiric antimicrobial therapy is crucial for the survival of sepsis patients [4,5]. Formerly, multidrug-resistant pathogens were found almost exclusively in nosocomial infections. However, community-acquired infections are now often caused by antibiotic-resistant bacteria (for example, extended-spectrum β-lactamase-producing Enterobacteriaceae, multidrug-resistant *Pseudomonas aeruginosa*, or methicillin-resistant *Staphylococcus aureus*) [14,15]. This striking change in epidemiology may explain why the initial therapy frequently includes a combination of different antimicrobial agents [16].

β-Lactams, including carbapenems, are the most commonly used antibiotics in the critical care setting [17]. Likewise, this antibiotic family constitutes the mainstay of empiric treatment in patients with severe sepsis or septic shock, whether administered alone or in combination with other antimicrobials. Carbapenems are more frequently prescribed in patients with nosocomial sepsis, although it is worth mentioning that one in five patients with community-acquired sepsis is treated empirically with a carbapenem. This may reflect the increase in multidrug-resistant gram-negative pathogens in the community [14]. Carbapenems might have been analyzed in conjunction with the rest of β-lactams. However, we decided to analyze them separately from other β-lactams because of the broader-spectrum, major role in empiric antibiotic therapy and the widespread use in the ICU.

Quinolones are used mainly in community-acquired infections and in combination therapy [18]. The extended use of quinolones in combination therapy in patients with severe community-acquired pneumonia may explain the increasing rate of quinolone resistance among nosocomial gram-negative pathogens [18,19].

Numerous studies have evaluated the likely superiority of combination therapy in patients with diverse types of infections. A French multicenter study of critical patients with acute peritonitis found no difference in the rate of therapeutic failure or length of antibiotic treatment when β-lactams were administered alone or in combination with aminoglycosides, concluding that aminoglycosides should be added only when an infection by *Pseudomonas *spp or *Enterococcus *spp is suspected [20]. Two randomized clinical trials found no benefits of combination therapy over monotherapy in patients with ventilator-associated pneumonia [21,22]. Moreover, in one trial, monotherapy was associated with lower rates of therapeutic failure, superinfection, and side effects [22].

Conversely, diverse studies have demonstrated lower mortality and length of stay in patients with pneumococcal bacteremia or with community-acquired pneumonia receiving combination therapy, including a β-lactam plus a macrolide or a quinolone, than in those receiving monotherapy [23-25]. In these studies, the benefits seem to be restricted to more-severe patients or to those in septic shock [18,23]. Conversely, a recent retrospective study concluded that, in bacteremia caused by gram-negative bacilli, combination therapy with β-lactams and fluoroquinolones was associated with a reduction in 28-day crude mortality only among less severely ill patients [7].

Two meta-analyses of studies performed in patients with gram-negative bacteremia or sepsis found no benefit of combination therapy over monotherapy, except when bacteremia was caused by multidrug-resistant bacteria or *Pseudomonas *spp [26,27]. Moreover, higher rates of side effects (mainly nephrotoxicity) were reported in the group of patients treated with β-lactam antibiotics plus aminoglycosides. More recently, a meta-analytic/meta-regression study that included 50 studies found that combination antibiotic therapy improves survival, particularly in septic shock patients, but may be harmful to less severely ill patients [28].

Nevertheless, few data are available about the impact on the outcome of combination therapy in large cohorts of patients with severe sepsis or septic shock. A recent propensity-matched analysis concluded that, in patients with septic shock, the use of combination therapy with two or more antibiotics of different mechanistic classes was associated with lower 28-day mortality, shorter ICU stay, and lower in-hospital mortality [10].

Our results confirm that combination therapy, including two or more antimicrobials with different mechanisms of action (β-lactams in combination with aminoglycosides, fluoroquinolones, or macrolides/clindamycin), administered within the first 6 hours of sepsis presentation is an independent protective factor against in-hospital mortality. Interestingly, severity of illness measured by APACHE II score, basal lactate levels, and the presence of hemodynamic failure did not differ between patients receiving DCCTs and those receiving non-DCCTs.

The choice of empiric antimicrobial therapy is based on the clinical presentation of the infection, the characteristics of the patient, the local ecology, and previous antibiotic exposure. Reducing the antibiotic pressure and side effects are the main reasons for choosing monotherapy. Conversely, the main reason for prescribing combination therapy for critically ill sepsis patient is to broaden the antimicrobial spectrum in an attempt to ensure the coverage of all likely pathogens. Our results permit us to speculate that the synergistic mechanisms of different antimicrobial combinations, or the immunomodulatory effects described with macrolides and quinolones, may be of clinical transcendence in patients with severe sepsis or septic shock [29-31].

Our study has several limitations. First, a major limitation in our study is the lack of microbiology data due to the initial study design. Accordingly, no data are available on antibiotic susceptibility, appropriateness of antimicrobial therapy, or the presence of bacteremia. Appropriate antimicrobial therapy based on culture results was an important determinant of survival in a large cohort of patients with severe sepsis [32].

Second, because of the absence of microbiology data, we cannot explore whether the positive impact on mortality observed with DCCTs is related to a synergistic effect of two mechanistically distinct antibiotics or a broader range of coverage with two or more agents.

Third, we did not evaluate source control and other important measures included in the Surviving Sepsis Campaign care bundles.

Fourth, this is a secondary analysis of an observational study. Nevertheless, properly designed observational studies with the appropriate analytic approach can provide valuable information on treatment effectiveness [4].

However, our study has also important strengths. We prospectively enrolled a large cohort of ICU patients with severe sepsis and septic shock in a short time and monitored them until death or hospital discharge, resulting in a homogeneous database with high quality-control measures that assure data validity [4]. Finally, our conclusions are strengthened by absence of immortal bias.

## Conclusions

β-Lactams, including carbapenems, are the mainstay of empiric therapy in patients with severe sepsis and septic shock. Carbapenems are more frequently prescribed in patients with nosocomial sepsis, although up to one in five patients with community-acquired sepsis is treated empirically with a carbapenem. Our study supports the hypothesis that early administration of antimicrobials with different mechanistic targets is associated with decreased in-hospital mortality. Our findings extend those of the propensity-matched analysis in patients with septic shock published by Kumar *et al. *[10] because we also included patients with severe sepsis, underlining the urgent need for well-designed randomized controlled trials to evaluate the clinical benefit of DCCTs in critically ill sepsis patients.

## Key messages

• β-Lactams, including carbapenems, are the antibiotics most usually used in the critical care setting.

• Although carbapenems are more frequently prescribed in patients with nosocomial sepsis, one in five patients with community-acquired sepsis is treated empirically with a carbapenem.

• Urologic focus of infection is associated with the lowest mortality rate in patients with severe sepsis or septic shock.

• In our series, the mortality rate was significantly lower in patients receiving DCCTs than in those receiving non-DCCTs.

• A DCCT in empiric therapy is a protective factor for mortality in patients with severe sepsis or septic shock.

## Abbreviations

APACHE II: Acute Physiology and Chronic Health Evaluation II; DCCT: different-class combination therapy; ICU: intensive care unit.

## Competing interests

The authors declare that they have no competing interests.

## Authors' contributions

As principal investigator, ADM had full access to all data in the study and takes responsibility for the integrity of the data and the accuracy of the data analysis. Study concept and design were performed by ADM, MLMG, RF, AA, JGM. RF completed the acquisition of data. Analysis and interpretation of data were performed by ADM and RF. Drafting of the manuscript was executed by ADM and JGM. Critical revision of the manuscript for important intellectual content was done by MLMG, COL, EP, MJLP, IML, ML, and AA. RF and AA carried out the Edusepsis General Coordination and Surviving Sepsis Campaign coordination by ML. All authors critically revised and approved the manuscript.
